# Cortical Thickness in Dementia with Lewy Bodies and Alzheimer's Disease: A Comparison of Prodromal and Dementia Stages

**DOI:** 10.1371/journal.pone.0127396

**Published:** 2015-06-10

**Authors:** Frederic Blanc, Sean J. Colloby, Nathalie Philippi, Xavier de Pétigny, Barbara Jung, Catherine Demuynck, Clélie Phillipps, Pierre Anthony, Alan Thomas, Fabrice Bing, Julien Lamy, Catherine Martin-Hunyadi, John T. O'Brien, Benjamin Cretin, Ian McKeith, Jean-Paul Armspach, John-Paul Taylor

**Affiliations:** 1 University Hospital of Strasbourg, Neuropsychology Unit, Neurology Service, Strasbourg, France; 2 University of Strasbourg and CNRS, ICube laboratory UMR 7357 and FMTS (Fédération de MédecineTranslationnelle de Strasbourg), team IMIS/Neurocrypto, Strasbourg, France; 3 University Hospital of Strasbourg, CMRR (Memory Resources and Research Centre), Strasbourg, France; 4 Institute of Neuroscience, Campus for Aging and Vitality, Newcastle University, Newcastle upon Tyne, United Kingdom; 5 University Hospital of Strasbourg, Hôpital de jour de gériatrie, Geriatry Service, Strasbourg, France; 6 Department of Psychiatry, University of Cambridge, Cambridge Biomedical Campus, Cambridge, United Kingdom; 7 University Hospital of Strasbourg, Neuroradiology Service, Strasbourg, France; Beijing Normal University,Beijing, CHINA

## Abstract

**Objectives:**

To assess and compare cortical thickness (CTh) of patients with prodromal Dementia with Lewy bodies (pro-DLB), prodromal Alzheimer's disease (pro-AD), DLB dementia (DLB-d), AD dementia (AD-d) and normal ageing.

**Methods:**

Study participants(28 pro-DLB, 27 pro-AD, 31 DLB-d, 54 AD-d and 33 elderly controls) underwent 3Tesla T1 3D MRI and detailed clinical and cognitive assessments. We used FreeSurfer analysis package to measure CTh and investigate patterns of cortical thinning across groups.

**Results:**

Comparison of CTh between pro-DLB and pro-AD (p<0.05, FDR corrected) showed more right anterior insula thinning in pro-DLB, and more bilateral parietal lobe and left parahippocampal gyri thinning in pro-AD. Comparison of prodromal patients to healthy elderly controls showed the involvement of the same regions. In DLB-d (p<0.05, FDR corrected) cortical thinning was found predominantly in the right temporo-parietal junction, and insula, cingulate, orbitofrontal and lateral occipital cortices. In AD-d(p<0.05, FDR corrected),the most significant areas affected included the entorhinal cortices, parahippocampal gyri and parietal lobes. The comparison of AD-d and DLB-d demonstrated more CTh in AD-d in the left entorhinal cortex (p<0.05, FDR corrected).

**Conclusion:**

Cortical thickness is a sensitive measure for characterising patterns of grey matter atrophy in early stages of DLB distinct from AD. Right anterior insula involvement may be a key region at the prodromal stage of DLB and needs further investigation.

## Introduction

Dementia with Lewy bodies (DLB) is the second most common form of dementia after Alzheimer's disease (AD), accounting for 15% to 20% of neuropathologically defined cases[1].Diagnostic classification of DLB is based on revised consensus criteria with core diagnostic features of DLB being(1) recurrent visual hallucinations, (2) cognitive fluctuations, and (3) spontaneous motor features of parkinsonism[1]. The presence of 2 or 3 of these core signs is sufficient for a diagnosis of probable DLB[1].However, distinguishing DLB from AD continues to be difficult because of overlapping clinical and neuropathological features between the two conditions. The accurate differentiation of DLB and AD, however, is particularly important as:1) the aetiological basis of both diseases is likely to be different[2];2) DLBs exhibit adverse sensitivity to neuroleptics; and 3) DLBs have a differing prognosis compared to AD[3],but yet a better response to cholinesterase inhibitors[4].

The diagnostic challenge becomes particularly salient in the early stages or prodromal stages (pro-DLB) of disease when the disease is present but cognitive impairments are not sufficient to lead to functional deficits in activities of daily living[5].In contrast to AD where there are significant advances in the classification and definition of prodromal AD (pro-AD),[6]the diagnostic classification of pro-DLB remains in its infancy although a prodromal phase of DLB has now been demarcated in DSM-V as mild neurocognitive disorder of Lewy body disease[7] and preliminary descriptions of pro-DLB criteria have recently been described[8];broadly, pro-DLB can be defined as those patients who meet the revised diagnostic criteria for DLB but instead of dementia[1], fit the criteria for mild cognitive impairment (MCI)[5].

Pro-DLB has been described with a different cognitive pattern from pro-AD[9, 10]: at the early stage of the disease, DLB patients have more visuospatial and letter fluency deficits than AD, and AD patients more memory storage impairment than DLB[10, 11]; findings which are in keeping with the cognitive profiles of AD and DLB patients when the dementia becomes manifest[12]although the neuropsychological pattern of pro-DLB has been reported as more heterogeneous than in pro-AD[11].

Nevertheless early identification of DLB, particularly in the prodromal phase (i.e. pro-DLB) will be highly relevant to the development and testing of future disease modifying treatments and thus there is urgent need to develop viable and sensitive biomarkers which can detect DLB in its early stages. Furthermore determination of early biomarkers in DLB are necessary to help guide the operationalization of future consensus criteria for pro-DLB[8].

Structural neuroimaging represents one potential biomarker area and, in particular, the metric of cortical thickness (CTh), which is an advanced and relatively novel method of structural image analysis. This approach allows for the quantification and regional distribution of cortical grey matter loss to be specifically examined which is in contrast to gyral or lobar volumetric studies which often combine grey matter and white matter within regional volumes. Previous cortical thickness studies in Dementia with Lewy Bodies at the stage of dementia (DLB-d) compared to Alzheimer's disease at the stage of dementia (AD-d)have reported thinning in the cingulate cortex, temporo-parieto-occipital areas, orbito-frontal cortex and insula[13, 14]. However the precise pattern of atrophy in pro-DLB is not known although one might hypothese posterior cortical changes might be a feature given: 1) Visuospatial dysfunction and the manifestation of visual hallucinations may be early features of DLB [9, 10, 15]; 2) There is tentative evidence from several studies using [18F]-fluoro-d-glucose (FDG) positron emission tomography (PET)[16]that patients with prodromal DLB symptoms have occipital hypometabolism[17].

Therefore the primary aim of this study was to investigate CTh patterns in subjects with pro-DLB and we report MRI patterns of CTh in subjects with DLB at the stage of MCI (pro-DLB) and established dementia (DLB-d), AD at the stage of MCI (pro-AD) and dementia (AD-d),as well as data from healthy elderly controls (HC).We included comparator disease groups to explore how cortical thinning may evolve from an early stage of disease (pro-DLB and pro-AD) to later established disease (DLB-d and AD-d); similarly inclusion of pro-AD group was also considered relevant as this would be the group from which pro-DLB would most likely need to be most distinguished from in clinical practise.

We hypothesised that in pro-AD, the pattern of cortical thinning would involve predominantly the temporal lobe, and parietal association cortices. In contrast, we expected that the pattern of cortical thinning in pro-DLB would be less diffuse involving predominantly posterior structures.

## Patients and Methods

### Ethics

The research was approved by the local ethics committee from SXB named "Comité de Protection des PersonnesEst IV" and NCL named "NRES Committee North East Sunderland" and "NRES Committee North East Newcastle & North Tyneside 2". All subjects or, where appropriate, their nearest relative, provided written informed consent.

### Subjects, assessments and diagnosis

**Fig 1:**
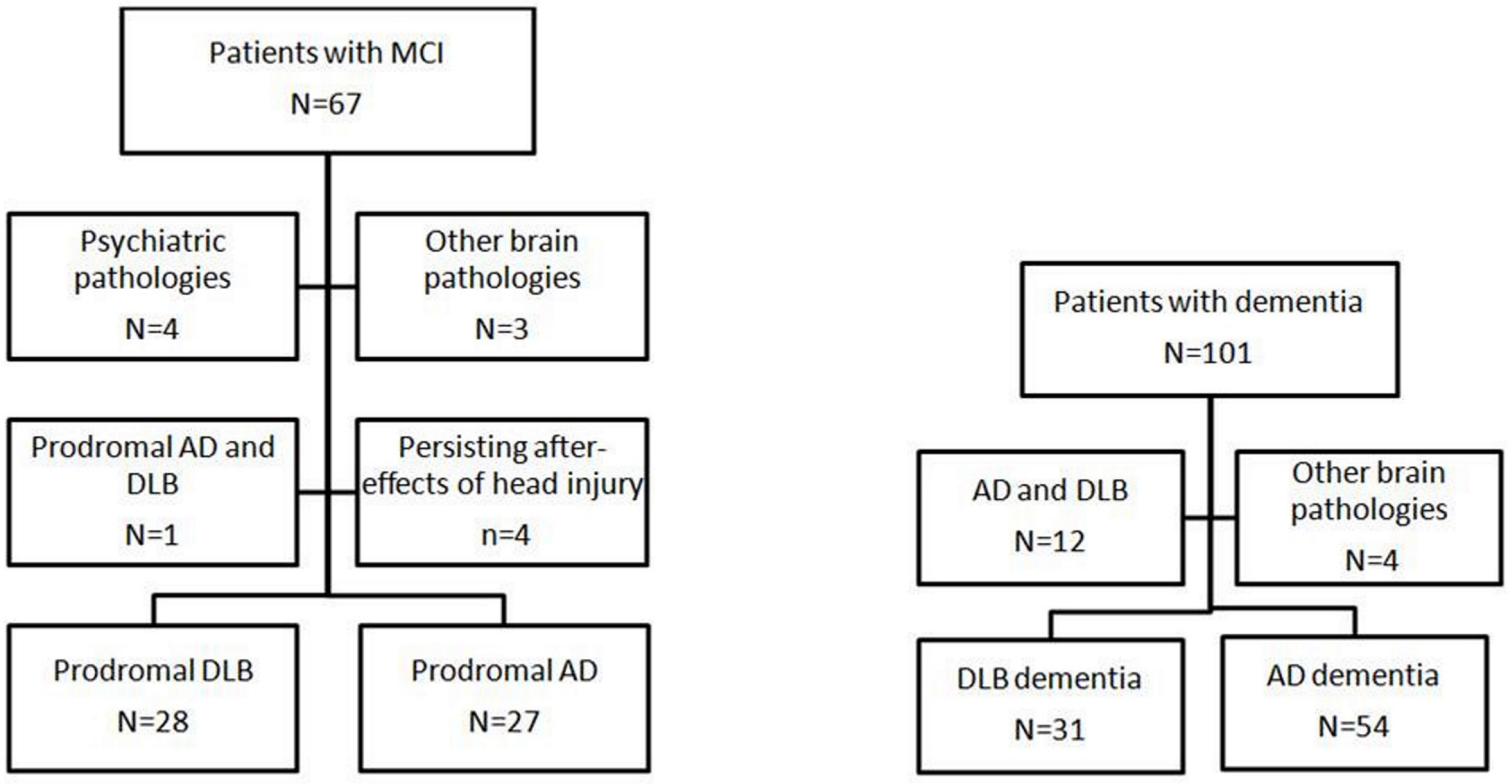
Flow Chart of the present study on cortical thickness in prodromal and dementia stages of Lewy body dementia and Alzheimer's disease. AD = Alzheimer’s disease; DLB = Dementia with Lewy bodies; MCI = Mild Cognitive Impairment. Prodromal DLB means patients with McKeith's criteria of DLB with cognitive impairment but without dementia. Psychiatric pathologies included two patients with depression, one with bipolar disorder, and one histrionic personality disorder; and one cognitive impairment due to severe sleep apnoea, one vitamin B12 encephalopathy, and one mitochondriopathy for patients with MCI. Other brain pathologies included one Parkinson's disease dementia, one DLB with primary Sjögren's syndrome, one with chronic brain autoimmune encephalitis and one dementia without evolution for more than 10 years, for patients with dementia

One hundred and sixty eight individuals suspected of DLB or AD over the age of 50 were recruited (see Fig 1: flow chart) from two European centres: 80were recruited from a community dwelling population of patients referred to local Old Age Psychiatry, Geriatric Medicine or Neurology Services from Newcastle upon Tyne (NCL); 88 were recruited from the tertiary Memory clinic (CMRR) of Strasbourg (SXB) including Neurology and Geriatric Medicine Services. Subjects underwent detailed clinical and neuropsychological evaluations. Common elements between centres included the assessment of motor parkinsonism with the Unified Parkinson’s Disease Rating Scale Part III (UPDRS-III)[18], the Clinician Assessment of Fluctuation (CAF)[19],the Mini-Mental State Examination (MMSE), the Clinical Dementia Rating scale (CDR), the trail making task A(TMTA) and B (TMTB). For TMT A and B, normative data from Tombaugh were used[20]. The neuropsychological evaluation of SXB included the Free and Cued Selective Reminding Tests (FC-SRT)for verbal memory, DMS-48 for visual recognition memory, forward and back ward Digit span, WAIS code for attention and speed processing, Frontal Assessment Battery (FAB) and phonemic fluencies for executive functions, semantic fluencies, Oral denomination 80 items (DO80) for language, the Rey-Osterrieth Complex Figure Test and Mahieux praxis evaluation. The neuropsychological evaluation of NCL was a comprehensive neuropsychological battery: the Cambridge Cognitive Examination (CAMCOG) as well as F-A-S test and semantic fluencies. For the purposes of this paper we report only those scales which were common to both centres (e.g. MMSE, TMTA and TMTB).

Pro-AD (n = 23),pro-DLB (n = 23), AD-d (n = 11), DLB-d (n = 3)(see Table 1)patients from SXBunderwent Cerebrospinal fluid (CSF) analysis including measurement of tau, phospho-tau, and amyloid-beta (1–42) (Innognetics’sInnotest, ELISA).Assessment of medial temporal atrophy on brain MRI was performed using the standardised Scheltensscale (5 categories, 0–4) with 0 corresponding to no atrophy[21]. Anaetiologic diagnosis of the neurocognitive disorder for each patient was made using Dubois’ criteria for pro-AD (n = 27, 26 from SXB, 1 from NCL) and AD-d (n = 54, 16 from SXB, 38 from NCL)[6],and McKeith’s criteria (probable DLB; two core symptoms) for DLB-d (n = 31, 3 from SXB, 28 from NCL)[1].Pro-DLB patients (n = 28, 26 from SXB, 2 from NCL) were defined as patients with MCI (Petersen criteria)[22], and a CDR of 0 or 0.5, and by McKeith's criteria (meeting probable DLB criteria except presence of dementia)[1] and this maps onto recent suggestions for potential pro-DLB criteria [8].Similarly33 aged healthy and cognitively intact (no MCI) subjects were recruited from among relatives and friends of subjects with neurocognitive disorders or volunteered via advertisements in local community newsletters inNCL and SXB. Exclusion criteria for participation in the study included contraindications for MRI, history of alcohol/substance misuse, evidence suggesting alternative neurological or psychiatric explanations for their symptoms/cognitive impairment, focal brain lesions on brain imaging or the presence of other severe or unstable medical illness.All patients had formal assessment of their diagnosis bythree independent expert clinicians (JPT, AT, FB for NCL and FB, BC, NP for SXB) and controls underwent similar clinical and cognitive assessments to patients to exclude any that may have had an occult MCI or dementia.Patients with concomitant AD and DLB i.e.meetingbothMcKeith's(for probable DLB) and Dubois’ criteria were also excluded (see Fig 1).

**Table 1:** Clinical and Demographic Features of Dementia with Lewy bodies patients, Alzheimer’s Disease Patients at the stage of MCI or prodromal and dementia, and healthy elderly controls.

		Pro-DLBN = 28	DLB-d N = 31	Pro-AD N = 27	AD-d N = 54	HC N = 33	Test statistic,P	Post *hoc* ^g^
**Age, years** ^a^		67.5 (9.2)	77.7 (6.9)	69.3 (7.8)	75.7 (9.4)	72.4 (10.4)	F = 7.184, P<.0001*	Pro-DLB<DLB-d and AD-dPro-AD<DLB-d and AD-d
**Education** ^b^ **1/2/3**		11/4/8	5/10/2	11/3/12	10/26/7	1/14/11	H = 7.465, P = .113	
**Gender (F/M)**		16/12	10/21	7/20	22/32	18/15	χ^2^ = 8.860, P = .065	
**Handedness (R/L)**		26/2	26/5	24/3	50/4	29/4	χ^2^ = 3.264, P = .515	
**MMSE score** ^a^		27.6 (2.1)	20.5 (4.6)	26.9 (1.9)	20.8 (3.6)	29.4 (0.9)	H = 119.397, P<.0001*	HC< AD-d, DLB-d and pro-ADPro-DLB<AD-d and DLB-dPro-AD<AD-d and DLB-d
**TMTA** ^c^ **impaired subjects**		60.7%	82.3%	32.0%	43.2%	0%	H = 21.791, P<.0001*	HC>DLB-d and pro-DLBPro-AD>DLB-d
**TMTB** ^c^ **impaired subjects**		71.4%	88.2%	44.0%	72.0%	0%	H = 27.536, P<.0001*	HC>DLB-d, AD-d, pro-DLB
**CDR sum of box 0/0.5/1/2/3**		2/26/0/0/0	0/0/22/8/1	1/26/0/0/0	0/0/42/12/0	33/0/0/0/0	H = 157.167, P<.0001*	HC< AD-d, DLB-d, pro-DLB and pro-ADPro-DLB<AD-d and DLB-dPro-AD<AD-d and DLB-d
**Parkinsonism** ^c^	**Rigidity0/1/2/3/4**	7/20/1/0/0	8/8/8/5/2	23/4/0/0/0	49/5/0/0/0	33/0/0/0/0	H = 84.874, P<.0001*	Pro-DLB>HC, pro-AD and AD-dDLB-d>HC, pro-AD and AD-d
	**Akinesia0/1/2/3/4**	10/14/3/1/0	3/13/12/3/0	23/4/0/0/0	40/12/2/0/0	31/2/0/0/0	H = 73.814, P<.0001*	Pro-DLB>HC, pro-AD and AD-dDLB-d>HC, pro-AD and AD-d
	**Tremor at rest0/1/2/3/4**	17/9/2/0/0	11/9/10/1/0	27/0/0/0/0	52/2/0/0/0	33/0/0/0/0	H = 63.608, P<.0001*	Pro-DLB>HC, pro-AD and AD-dDLB-d>HC, pro-AD and AD-d
**Hallucinations** ^d^		60.7%	90.3%	0%	1.8%	0%	χ^2^ = 119.071, P<.0001*	
**Fluctuations** ^d^		92.9%	90.3%	0%	22.2%	0%	χ^2^ = 102.417, P<.0001*	
**CAF** ^a^		3.5 (3.6)	6.8 (4.9)	0.0 (0)	1.0 (2.7)	0 (0)	H = 81.063, P<.0001*	Pro-DLB>HC, pro-AD and AD-dDLB-d>HC, pro-AD and AD-d
**RBD** ^d^		56.0%	56.6%	7.7%	3.7%	0%	H = 65.852, P<.0001*	Pro-DLB>HC, pro-AD and AD-dDLB-d>HC, pro-AD and AD-d
**Treatment** ^d^	**ChEI**	28.6%	83.8%	48.1%	81.5%	0.0%	χ^2^ = 72.997, P<.0001*	
	**Dopa**	28.6%	35.5%	0.0%	0.0%	0.0%	χ^2^ = 41.804, P<.0001*	
	**NL**	10.7%	22.6%	0.0%	1.9%	0.0%	χ^2^ = 20.177, P = .0001*	
**CSF** ^e^	**Abeta-42**	859.3 (336.7, 23)	796.3 (277.3, 3)	579.0 (287.4, 23)	525.8 (104.0, 11)	-	F = 5.194, P = .003*	Pro-DLB>AD-d and pro-AD
	**P-Tau**	43.6 (13.9, 23)	66.0 (1.7, 3)	93.7 (36.8,23)	99.5 (39.5, 11)	-	F = 14.111, P<.0001*	Pro-DLB<AD-d and pro-AD
	**Tau**	313.0 (286.3, 23)	430.7 (28.6, 3)	660.6 (355.4, 23)	679.0 (246.1, 11)	-	F = 6.266, P = .001*	Pro-DLB<AD-d and pro-AD
**Hippocampi atrophy** ^f^ **0/1/2/3/4**	**Left hippocampus**	14/10/2/2/0	5/12/9/4/1	5/16/4/2/0	4/18/15/15/2	27/5/1/0/0	H = 61.935, P<.0001*	HC<AD-d, DLB-d and pro-ADPro-DLB<AD-d and DLB-d
	**Right Hippocampus**	14/10/4/0/0	7/9/11/3/1	5/14/7/1/0	4/16/19/13/2	22/8/2/1/0	H = 50.544, P<.0001*	HC<AD-d, DLB-d and pro-ADPro-DLB<AD-d and DLB-d

^a^ Mean (standard deviation).

^b^Education: 1 = before High School, 2 = High School, 3 = University.

^c^ As rated on Unified Parkinson's Disease Rating Scale^15^.

^d^Percentage.

^e^ Mean (standard deviation, number of patients tested).

^f^ according to Scheltens et al.,JNNP, 1992.

^**g**^Tukey post-hoc test for ANOVA (F), Mann-Whitney post-hoc test on SPSS (H). CAF = Clinician Assessment of Fluctuation; CDR = Clinical Dementia Rating; ChEI = CholinEsteraseInhbitor; Dopa = L-Dopa or dopaminergic agonists; MMSE = Mini-Mental Status Examination; NL = Neuroleptics (only clozapin or quetiapin); N = number; RBD = Rapid Eye Movement sleep Behaviour Disorder; TMTA = Trail Making Test A; TMTB = Trail Making Test B; UPDRS = Unified Parkinson's Disease Rating Scale.

### MRI data acquisition

Subjects from NCL and SXB underwent T1 weighted MR scanning on a 3T MRI systemwithin 2 months of the study assessment. NCLused an 8 channel head coil (InteraAchieva scanner, Philips Medical Systems, Eindhoven, Netherlands) and SXB a 32 channel head coil (Veriosyngo MR B17, Siemens magnetom). The sequence was a standard T1 weighted volumetric sequence covering the whole brain (3D MPRAGE, sagittal acquisition, 1 mm isotropic resolution). 3D T1 of NCL had a matrix size of 240 (anterior-posterior) x 240 (superior-inferior) x 180 (right-left), a repetition time (TR) = 9.6ms, an echo time (TE) = 4.6ms, and a flip angle = 8°. 3DT1 of SXB had matrix size of 192 (anterior-posterior) x 192 (superior-inferior) x 176 (right-left), a repetition time (TR) = 1900ms, an echo time (TE) = 2.53ms and a flip angle = 9°. The acquired volume was angulated such that the axial slice orientation was standardised to align with the AC-PC line.

### Imaging processing

Estimates of CTh were performed from cortical surface reconstructions computed from T1 weighted images using FreeSurfer (v. 5.1, http://surfer.nmr.mgh.harvard.edu/). The technical aspects of these methods have been described,in detail, elsewhere[23, 24]. In brief, the processing stream involved intensity non-uniformity correction, Talairach registration, removal of non-brain tissue (skull stripping), white matter (WM) and subcortical grey matter (GM) segmentation, tessellation of the GM-WM boundary then surface deformation following GM-CSF intensity gradients to optimally place GM-WM and GM-CSF borders[23, 24]. Once cortical models were generated, surface inflation, transformation to a spherical atlas and parcellation of the cerebral cortex into regions based on gyraland sulcal structure were carried out[25]. This technique used both intensity and continuity information from the entire 3D MR volume in the segmentation and deformation procedures to produce representations of CTh, calculated as the closest distance from the GM-WM to GM-CSF boundaries at each vertex on the tessellated surface[26].CTh measures were mapped to the inflated surface. All images were then aligned to a common surface template and smoothed with a 20mm full width at half maximum (FWHM) surface based Gaussian kernel.

Visual inspection of images at each step of the FreeSurfer processing stream were carefully carried out (by FB and SJ.C) to ensure accurate Talairach transformations, skull strips, deep GM and white/pial surface generation and tissue classifications. During this procedure,pial and/or WM surface errors were initially identified in 47scans. Manual correctionswere then performed on these scans such as removal of dura mater and/orthe applicationof a set of WM control points as required, before regeneratingthe pialor WM surfaces or both.Modification to the processing stream resulted in successful cortical surface regeneration of31 scans. However, the remaining 16scans (1 healthy subject, 5AD-d, 1 pro-AD, 2 DLB-d and 7 pro-DLB), still exhibited significant pial or WM surface errors and were therefore excluded. The dataset for subsequent CTh analysis therefore comprised of 33 controls, 54 AD-d, 31 DLB-d, 27 pro-AD and 28 pro-DLB.

### Statistical analysis

The Statistical Package for Social Sciences software (SPSS ver. 21.0.0.0, http://www-01.ibm.com/software/analytics/spss/) was used for further statistical evaluation as required. Where appropriate, differences in demographic and clinical data were assessed using parametric (ANOVA, t-tests) and non-parametric tests (Kruskall-Wallis H, Mann-Whitney U). Post-*hoc*analyses employedTukey and Mann-Whitney U for ANOVA and Kruskall-Wallis tests respectively.For categorical measures, χ^2^ tests were applied. For each test statistic, a probability value of <0.05 was regarded as significant.

#### Cortical thickness

Regional CTh between groups were examined on a vertex-wise basis using the general linear model (GLM), performed with the QDEC software (http://surfer.nmr.mgh.harvard.edu/fswiki/Qdec).

CTh was modelled as a function of group, controlling for effects of age and where applicable ‘MRI site sequence’ as nuisance covariates. CTh = _1_Group1 + β_2_Group2 + β_3_ Age+ β_4_Sequence +μ + ε (where μ is a constant and ε is error). Contrasts of interest were calculated using two-tailed t-tests between the group estimates β_1_ and β_2_. Surface maps showing significant differences between groups were then generated. Effects of CTh on global cognition(MMSE) were investigatedwith age and MRI site sequence as nuisance variables. CTh was modelled as a function of covariate of interestCTh = β_1_MMSE+β_2_Age + β_3_Sequence+μ+ ε. Contrasts of interest were calculated from the estimate β_1_, and whether β_1_ was significantly different from zero. For all statistical analyses, a false discovery rate approach (FDR) was used.

## Results

### Subject characteristics

The demographic data for patients and healthy subjects are summarised in Table 1. Subject groups were well matched for education, gender and handedness. Patients with prodromal disease were younger than patients with dementia.Of the patients with pro-AD 9 presented with amnestic MCI single domain and 18 with amnestic MCI multiple domain.Of the patients with pro-DLB 2 presented with amnestic MCI multiple domain, 13 with non-amnestic MCI single domain and 13 with non-amnestic MCI multiple domain. For pro-AD and pro-DLB, MMSE scores were similar, and higher than in AD-d and DLB-d groups. For TMTA, patients with DLB (pro-DLB or DLB-d) were more impaired than the healthy control group. DLB-d patients were also more impaired than pro-AD. For TMTB, patients with dementia (DLB-d and AD-d), and pro-DLB were more impaired than healthy controls.As expected, CDR scores were higher in the dementia groups than in the prodromal groups.Bothpro-DLB and DLB-d had significantly higher motor parkinsonism (UPDRS III scores), higher CAF score, and higher prevalence rate of REM sleep behaviour disorder (RBD) than the other groups. Patients with DLB (prodromal or dementia) were also on dopaminergic treatment and DLB-d patients had the highest usage of neuroleptics (clozapine and quetiapine) compared to other groups.Across AD and DLB, patients with dementia weremore often taking cholinesterase inhibitors than prodromal patients.Patients with AD (prodromal and dementia) had a greater number ofabnormal CSF biomarkers than DLB (prodromal and dementia). Visual rating of atrophy on MR scans found overall patients with dementia had greater hippocampal atrophy than controls, whilst DLB-pro had less atrophy than either AD or DLB-d.

### Comparison of pro-DLB and healthy subjects

**Fig 2:**
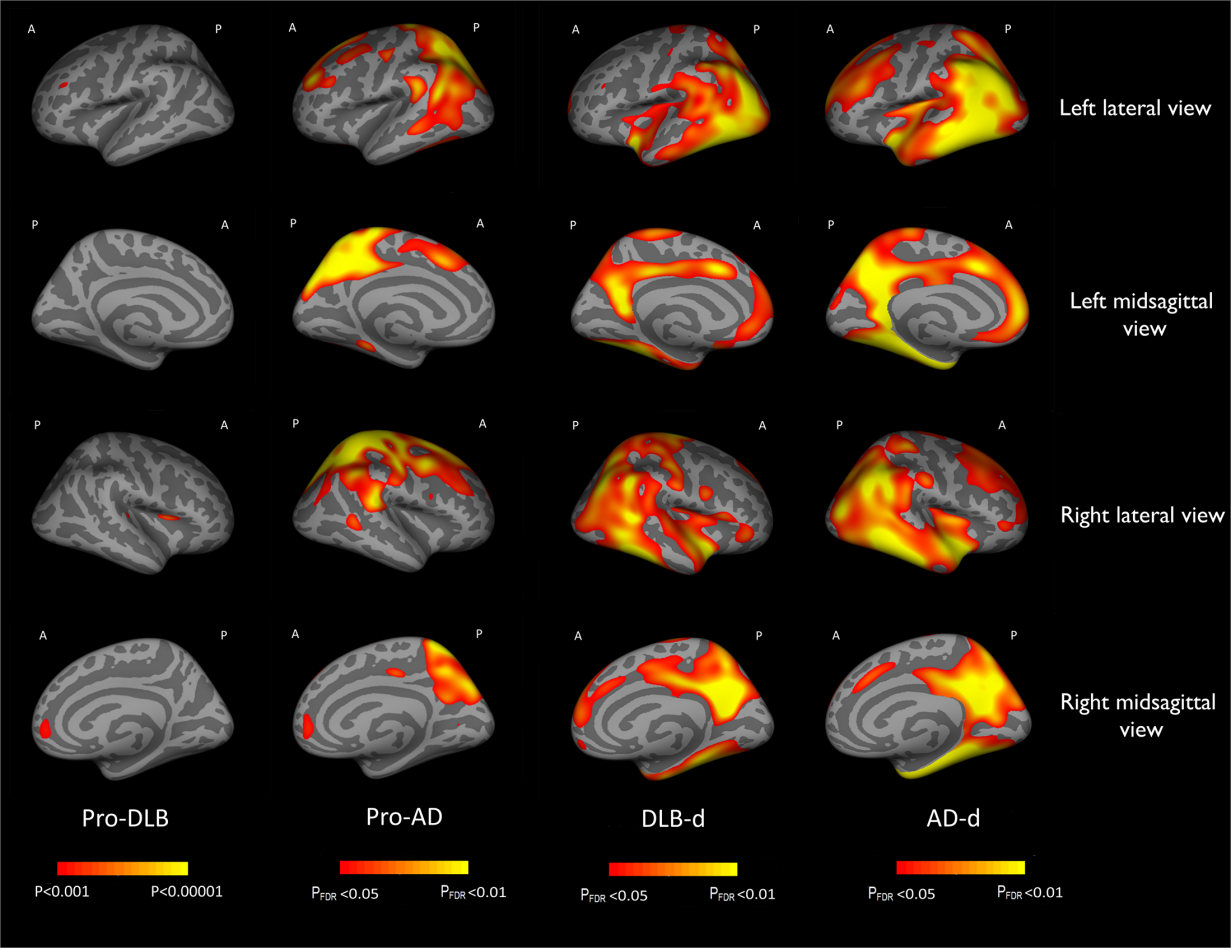
Cortical thinning patterns in pro-DLB, pro-AD, DLB-d and AD-d compared to healthy older controls (A = Anterior, P = Posterior, FDR = False Discovery Rate).

Areas of cortical thinning in pro-DLB compared to healthy subjects are shown in Fig 2 (first column), (n = 61, df = 58, p<0.001, uncorrected) and Table 2(A). Two clusters were noted on the right hemisphere: right insula/pars opercularis and in the right medial orbitofrontal regions. No difference in cortical thinning was found post FDR correction (<0.05).

**Table 2:** Location and peak vertex significance of cortical thinning in pro-DLB, DLB-d, pro-AD and AD-d compared to healthy subjects as well as pro-DLB relative to pro-AD, and DLB-d compared to AD-d.

	x	y	z	NVtxs	Area (mm2)	-log_10_ *P*
**(a) Pro-DLB vs. healthy subjects** *						
Left rostral middle frontal	-43	32	25	92	51	3.25*
Rightinsula/parsopercularis	36	10	13	492	154	4.66*
Right medial orbitofrontal	14	46	0	319	182	3.32*
Right superior temporal	47	-26	6	63	29	3.18*
**(b) Pro-AD vs. healthy subjects**						
Left superior parietal	-30	-52	51	35551	16920	5.02
Left superior frontal	-17	25	53	7510	4370	4.08
Left supramarginal	-47	-41	27	1933	904	3.66
Left parahippocampal	-34	-38	-12	665	303	2.94
Left precentral	-35	-18	36	814	307	2.86
Left inferior temporal	-48	-42	-20	1234	780	2.81
Left caudal middle frontal	-36	17	50	1609	982	2.58
Left lateral orbitofrontal	-21	12	-19	238	112	2.46
Right superior parietal	28	-44	61	47740	22788	6.01
Right middle temporal	-60	-50	3	678	363	2.68
Right posterior cingulate	12	-21	38	354	128	2.64
Right medial orbitofrontal	14	44	-1	395	211	2.15
Right pars opercularis	46	5	19	71	31	1.90
**(c) DLB-d vs. healthy subjects**						
Left fusiform	-39	-63	-17	60864	30466	7.17
Left medial orbitofrontal	-9	15	-16	4185	2471	2.78
Left superior frontal	-9	34	52	182	104	1.82
Left postcentral	-39	-22	-22	141	53	1.81
Left rostral middle frontal	-44	30	25	44	25	1.76
Right precuneus	7	-60	20	72400	34876	6.65
Right superior frontal	13	48	34	4013	2353	3.03
Right precentral	44	4	23	641	311	2.63
Right medial orbitofrontal	7	31	-17	180	137	1.86
**(d) AD-d vs. healthy subjects**						
Left entorhinal	-20	-17	-26	91291	47438	10.48
Left postcentral	-40	-26	48	647	248	1.72
Right entorhinal	22	-8	-30	71015	35943	8.29
Right superior frontal	16	45	38	10167	6104	2.79
Right precentral	16	-20	71	1280	546	2.14
**(e) pro-DLB vs. Pro-AD**						
Left superior parietal	-28	-51	38	26483	12123	6.52
Left parahippocampal	-21	-19	-25	363	134	3.20
Left supramarginal	-58	-34	28	340	175	2.46
Left precentral	-26	-13	55	223	102	2.41
Left lingual	-12	-58	-3	318	157	2.27
Left lateral occipital	-43	-67	-3	264	150	2.26
Left fusiform	-39	-72	-17	386	272	2.24
Right inferior parietal	33	-57	42	22042	10237	7.81
Right insula	36	9	13	1049	333	-5.66
Right precentral	26	-13	50	1428	607	3.71
Right precuneus	9	-59	45	270	103	2.46
**(f) DLB-d vs. AD-d**						
Left entorhinal	-20	-12	-28	221	89	4.88

Table depicts anatomical region, FreeSurferTalairach coordinates, number of vertices within each cluster (NVtxs), surface area (Area) (mm2) and peak significance expressed as a—log10P value (e.g.,-log10P = 3 corresponds to p = 0.001…etc). All results are significant (p<0.05 FDR corrected) except where noted by asterisk (*) which were uncorrected.

### Comparison of pro-AD and healthy subjects

Areas of cortical thinning in pro-AD compared to healthy older individuals are shown in Fig 2 (third column, n = 63, df = 59, p<0.05, FDR corrected) and Table 2(B). In pro-AD, cortical thinning was diffuse and involved temporal, parietal and frontal lobes. The most significant areas affected were confined to the parietal lobes.

### Comparison of DLB-d and healthy subjects

Regions of cortical thinning in DLB compared to healthy subjects are presented in Fig 2 (second column, n = 64, df = 60, p<0.05, FDR corrected) and Table 2(C). In DLB-d, there was generally less cortical thinning than in AD-d when compared to healthy subjects. Significant areas involved were the bilateral temporo-parietal junction, insula, cingulate and orbitofrontal cortices, lateral occipital lobes and superior frontal and anterior cingulate.

### Comparison of AD-d and healthy subjects

Cortical thinning in AD-d compared to healthy older subjects are represented in Fig 2 (fourth column, n = 87, df = 83, p<0.05, FDR corrected) and Table 2(D). In AD-d, cortical thinning was bilaterally diffuse and involved large areas of the temporal, parietal, frontal and occipital lobes. The most significant areas affected included the entorhinal cortices, parahippocampal gyri and parietal lobes.

### Comparison of pro-AD and pro-DLB

**Fig 3:**
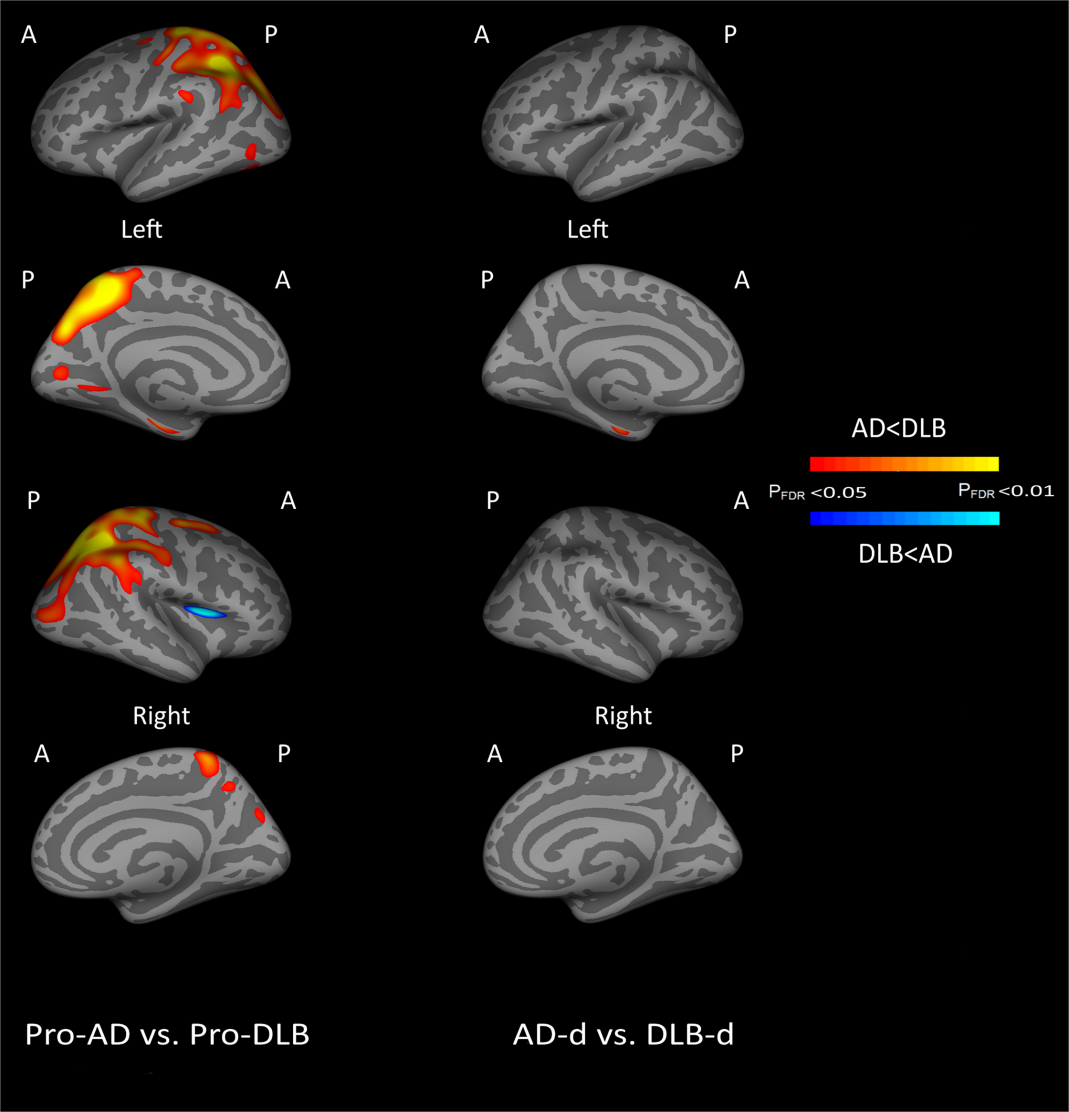
Patterns of Cortical thinning between Pro-AD and Pro-DLB and between AD-d and DLB-d (A = Anterior, P = Posterior, FDR = False Discovery Rate).

Regions of cortical thinning comparing pro-AD and pro-DLB are displayed in Fig 3 (first column, n = 55, df = 51, p<0.05, FDR corrected) and Table 2(E). In pro-DLB, there was generally less cortical thinning than in pro-AD. The most significant areas of thinning for pro-AD relative to pro-DLB were located in parietal lobes and left parahippocampal gyri, while for pro-DLB compared to pro-AD this was confined to the right insula and pars opercularis.

### Comparison of AD-d and DLB-d

Comparing AD and DLB, areas of significant cortical thinning (Fig 3 second column, n = 85, df = 81, p<0.05, FDR corrected, Table 2F) were observed in AD-d compared to DLB-din the left entorhinal cortex.

### Cortical thickness and cognition

**Fig 4:**
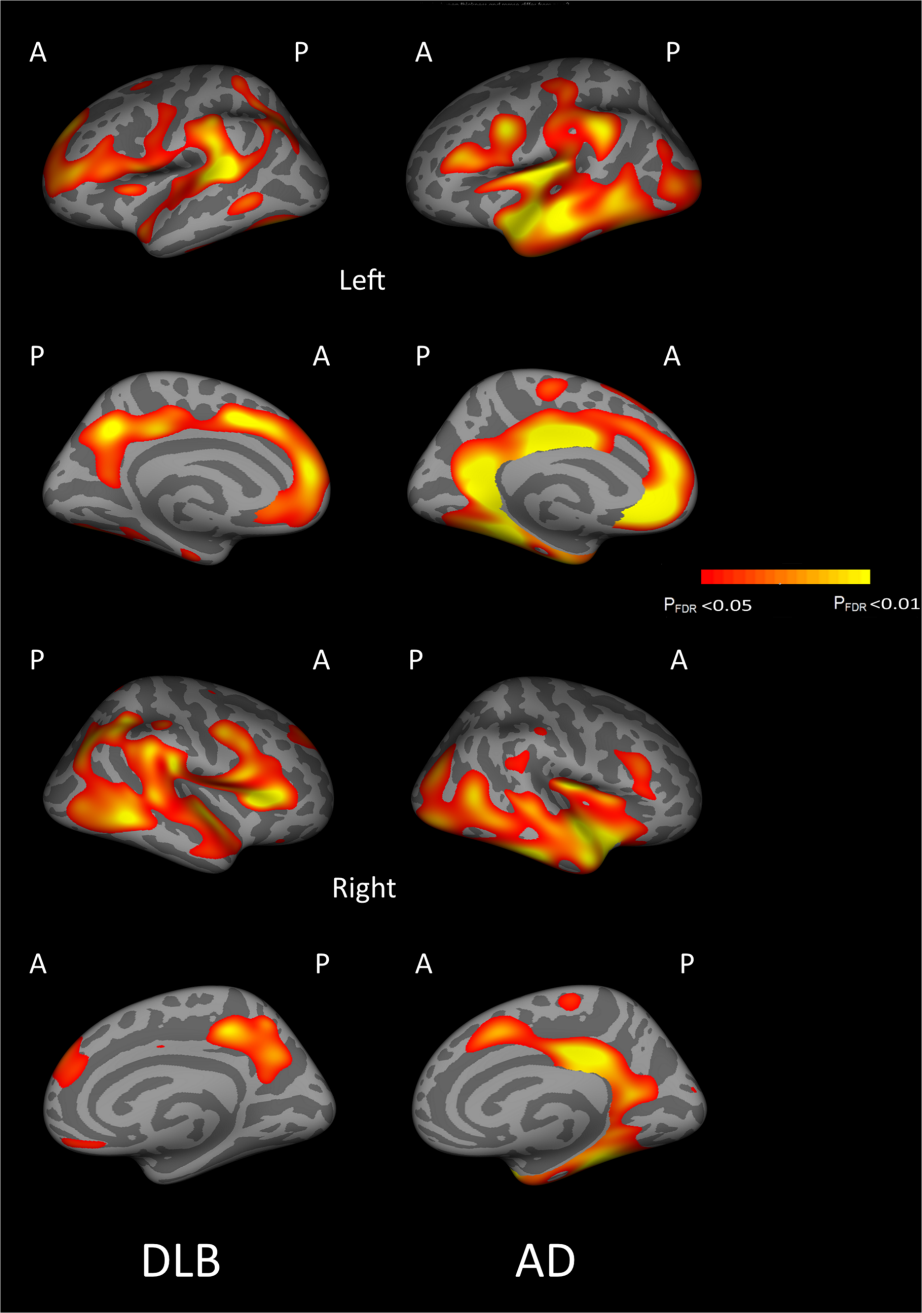
Correlations between cortical thickness and MMSE for dementia with Lewy bodies (pro-DLB and DLB-d: DLB) and Alzheimer's disease (pro-AD and AD-d: AD) patients (A = Anterior, P = Posterior, FDR = False Discovery Rate).

We did not find an association between CTh and MMSE performance in pro-DLB (n = 28, df = 26, p>0.05 FDR corrected).However in DLB-d subjects there was a positive association between MMSE and CThin widespread right hemispheric cortical areas including the pars triangularis, superior frontal, medial orbitofrontal, middle temporal, supramarginal, inferior parietal regions as well as precuneus, pars opercularis and insula (n = 31, df = 27, p<0.05 FDR corrected).In combined DLB (pro-DLB and DLB-d, Fig 4) there was a positive correlation found in the bilateral temporal, parietal and insula, left cingulate, right isthmus and posterior cingulate, bilateral rostral middle frontal and superior frontalgyri, left medial orbitofrontal and right lateral orbitofrontal(n = 59, df = 55, p<0.05 FDR corrected).No association was observed in pro-AD (n = 27, df = 23, p>0.05 FDR corrected).In AD-d subjects there was a positive relationship with MMSE and CTh in the cingulate cortex (rostral anterior, posterior, isthmus)and superior frontal regions of the left hemisphere (n = 54, df = 50, p<0.05 FDR corrected). For combined AD (pro-AD and AD-d, Fig 4), a positive association was also identified in the left temporal lobe, cingulate (isthmus, posterior, rostral anterior), precuneus, fusiform, supramarginal, precentral and rostral middle frontal (n = 81, df = 77, p<0.05 FDR corrected).

## Discussion

We report distinct, yet differing patterns of cortical thinning in pro-DLB, DLB-d, pro-AD and AD-d when compared to a group of healthy subjects. Pro-DLB was characterised by discrete cortical thinning in the right anterior insula and adjacent pars opercularis. Pro-AD was characterised by widespread cortical thinning in parietal lobe, and aspects of frontal and temporal lobes. The CTh comparison of the pro-DLB and pro-AD showed more cortical thinning in pro-AD in the parietal lobes and the parahippocampal regions. Only one region was thinner in pro-DLB than in pro-AD patients: the right anterior part of insula and the adjacent pars opercularis.

Cortical thinning was evident in the temporoparietal junction, parts of the temporal lobes including parahippocampal regions, bilateral insula, cingulate cortices, lateral part of the occipital lobes, and superior frontal and orbitofrontal cortices in DLB-d when compared to healthy controls. These findings were consistent with previous reports using cortical thickness and Voxel-Based Morphometry(VBM)[14, 27].In pro-DLB compared to healthy controls, there was evidence of cortical thinning in right frontal and insula regions, although this was only evident in uncorrected comparisons suggesting that cortical thinning, certainly in the prodromal stage of DLB is relatively mild.

AD-d was characterised by cortical thinning in confluent areas of parietal and temporal lobes, and a significant part of frontal and occipital lobes. The CTh comparison of the DLB-d and AD-d showed more cortical thinning in AD-d patients in left entorhinal cortex and this observation is in line with cortical thinning patterns reported in other independent DLB-d and AD-d datasets from the NCL group[14]. Furthermore this also aligns with other studies which have noted that DLB-d patients when compared to AD-d patients tend to haveless atrophy in the medial temporal regions particularly on the left[27], and the anterior part[28], but not in the posterior cortex[29].Howevercomparisons involving AD-d were unbalanceddue to the larger sample size, and therefore may incur increased type I errors and assuch,these results should be interpreted more tentatively.

In pro-AD cortical thinning was mainly seen in the parietal cortices; this is consistent with previous findings using freesurferwhich have shown parietal involvement in the early phase of AD but not the MTL; furthermore this pattern of atrophy has been demonstrated MCI patients with an AD trajectory[30],although it is notable that VBM has been shown to find more MTL atrophy than parietal with the same patients[31]. However from aCTh perspective, analysis with freesurfer can only partially assess the MTL and cannot be used assess hippocampal volumes. Nevertheless we confirmed on visual atrophy grading greater MTL atrophy in our AD-d cohort compared to DLB-d cohort in line with previously published data[14, 32].

Correlations between MMSE and CTh in DLB (pro-DLB and DLB-d), and in AD (pro-AD and pro-DLB) showed key regions associated with cognitive decline. However, no correlation was found with prodromal patients, pro-AD or pro-DLB; this is likely to be a function of less atrophy in prodromal patients and limits on the range of MMSE scores in these groups and lack of sensitivity of the MMSE to subtle cognitive deficits.

A key finding was the observationthat right anterior insula was thinner in pro-DLB and this thinning became more manifest at the dementia stage. In comparison, in pro-AD insular thinning was not evident;this is not unexpected as this region is not a part of the cortical thinning signature in early AD[33]. However with disease progression in AD where there is markedcellular loss, there is evidence of widespread cortical thinning which included insular areas (Fig 2). Thus insula thinning appears to be a feature of pro-DLB and not pro-AD but with time this difference becomes less apparent once dementia manifests. The thinning of the anterior insula was also associated with lower MMSE in the DLB-d and combined DLB-d and pro-DLB cohort. The insula isinvolved in integrating somatosensory, autonomic and cognitive-affective information to guide behaviour[34], and specifically the anterior insula has been described as part of a ‘salience network’ due to its consistent activation during cognitively demanding tasks, and the ability of this network to switch between brain networks involved in cognition, including the central executive and default-mode network[35].Interestingly, the anterior insular has certain specific neurons namely the Von Economo neurons (VENs), located in layer 5 of the cortex with a predominance in the right hemisphere, the same region we have found thinner in pro-DLB[36].Because of the larger size of VENs compared to pyramid neurons, they are purported be involved in the fast assessment of complex situations[36] and the 'salience network'[37] and thus it might been hypothesized that deficits in this region might be pertinent to the cognitive slowing and attentional deficits which typify DLB. Certainly abnormal resting state functional connectivity encompassing areas such as the right insula and right frontal operculum has been previously observed in DLBpatients with cognitive fluctuations[38].Interestingly, a recent voxelwisemeta-analysis on cortical atrophy of DLB patients at the stage of dementia found a bilateral insula atrophy[39]. Moreover, we recently described a right anterior insula atrophy of patients with cognitive neurodegenerative diseases and hallucinations[40].

Our study has somelimitations.Perhaps most salient is the fact thatwhilst there is an increasing recognition that DLB has a prodromal state there is no consensus,as yet, regarding itsclinical definition. In the absence of this we have utilised a combination of pre-existing criteria for MCI and DLB criteria; however it is unknown whether these criteria are optimal for prodromal DLB and we have no neuropathological confirmation of the diagnoses of any of our patients at present. However, we have used McKeith's criteria which has excellent specificity (more than 95%) [41, 42] when compared to gold standard neuropathological diagnosis.In addition, we also excluded other pathologies such as psychiatric illness, other neurological diseases, and also co-occurrence ofAD and DLB (see flow chart). Furthermore more than 50% of our DLB patients have RBD, which improves the specificity of the diagnosis[43].We also systematically looked for discrete clinical symptoms such as anosmia/hyposmia, constipation, and other autonomic features (data not shown)[44],as these have been previously demonstrated to improve the diagnostic specificity of the prodromal DLB patients[44].Another limitation is the cross-sectional nature of our study; longitudinal studies as in AD will be necessary for themapping the disease trajectory for patients in prodromal stage of DLB and how cortical thinning patterns evolve in DLB.

Another challenge in our study was the relatively modest number of patients studied and they spanned a range of disease severities.From a technical perspective, it could also be argued that a drawback of our study was the fact that data was collected from two sites with differing imaging protocols. However we controlled where possible for this in our analyses thus minimising this potential confound.Lastly, a possible future analysiswould be to examinethe reliabilityof the prodromal results for validation purposesusingresampling/subsampling techniques although this would require larger cohorts.

In conclusion, our data suggest that cortical thickness may be a sensitive measure for characterising grey matter atrophy in early stages of DLB and its variance with patterns of grey matter loss in early AD. In this context it may have a valuable role in delineating between prodromal states of DLB and AD, as well as helping shapefuture diagnostic criteria for prodromal DLB.
